# Supplementary figures and images for: Effects of Cadmium Stress on Tartary Buckwheat Seedlings
Source: Plants (Basel). 2024 Jun 14;13(12):1650. doi: 10.3390/plants13121650 (PMC11207290; doi:10.3390/plants13121650)

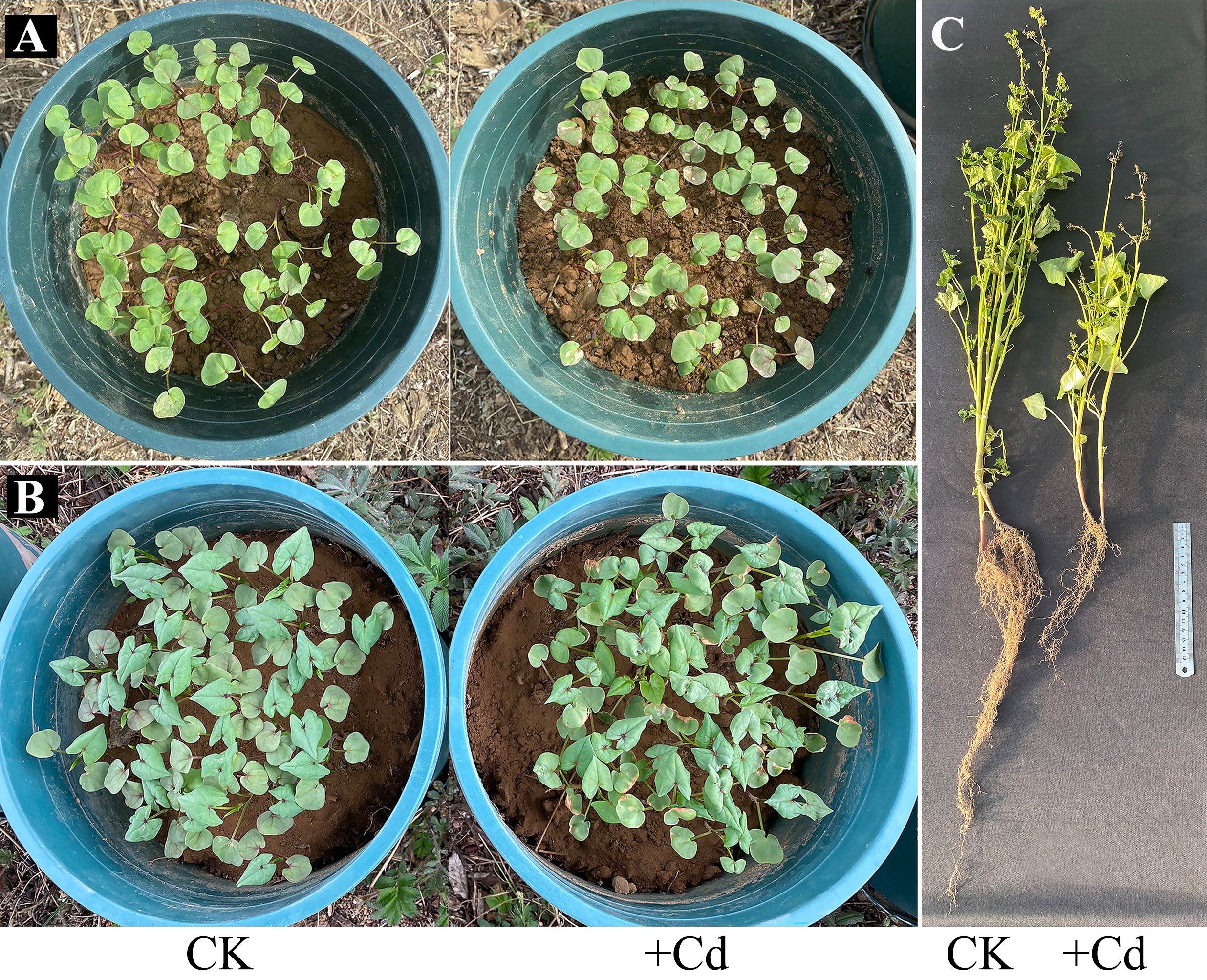

Supplement: Supplementary file 1 [file plants-13-01650-s001.zip › Fig S1.tif]

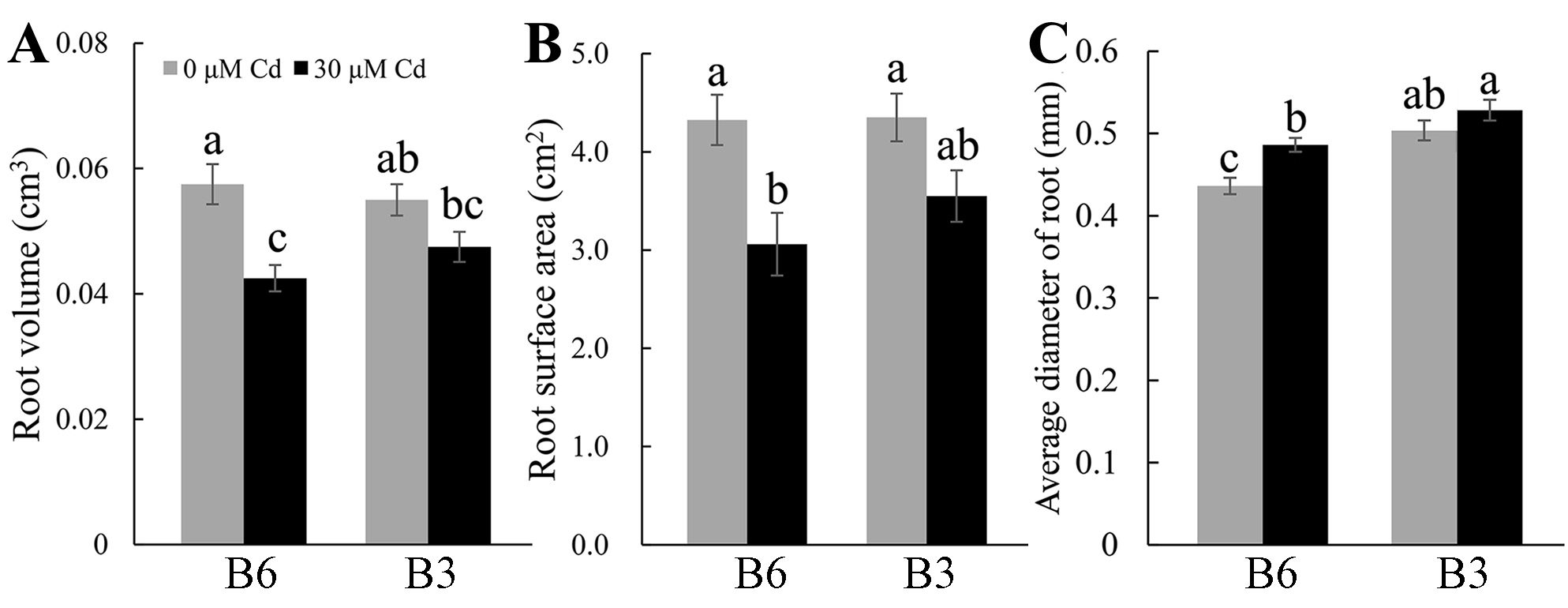

Supplement: Supplementary file 1 [file plants-13-01650-s001.zip › Fig S2.tif]

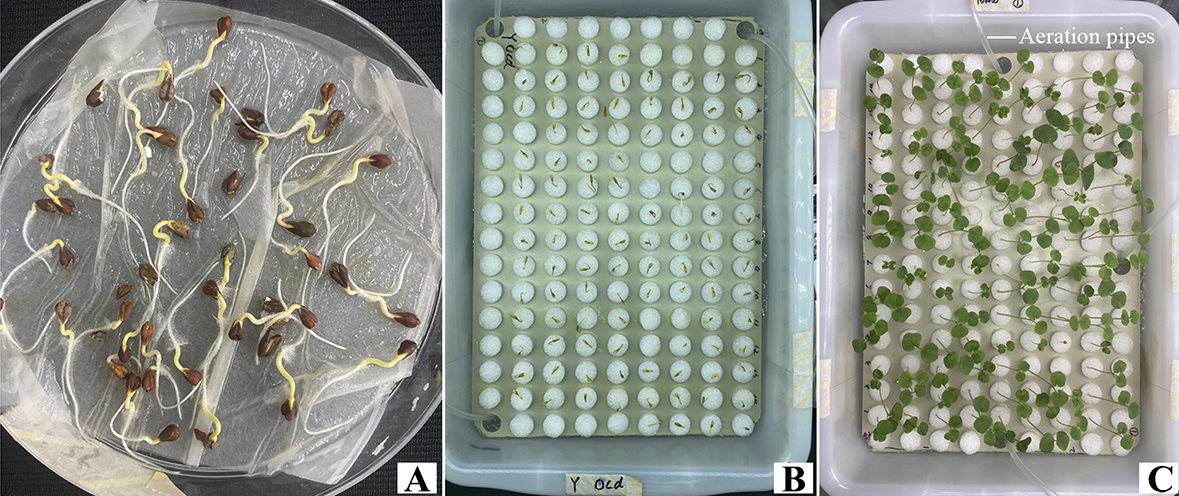

Supplement: Supplementary file 1 [file plants-13-01650-s001.zip › Fig S3.tif]
